# Challenges to conducting research with older people living in nursing homes

**DOI:** 10.1186/1471-2318-9-38

**Published:** 2009-08-24

**Authors:** Sue Hall, Susan Longhurst, Irene J Higginson

**Affiliations:** 1 King's College London, Department of Palliative Care, Policy & Rehabilitation, Weston Education Centre, Cutcombe Road, London SE5 9RJ, UK

## Abstract

**Background:**

Although older people are increasingly cared for in nursing homes towards the end of life, there is a dearth of research exploring the views of residents. There are however, a number of challenges and methodological issues involved in doing this. The aim of this paper is to discuss some of these, along with residents' views on taking part in a study of the perceptions of dignity of older people in care homes and make recommendations for future research in these settings.

**Methods:**

Qualitative interviews were used to obtain the views on maintaining dignity of 18 people aged 75 years and over, living in two private nursing homes in South East London. Detailed field notes on experiences of recruiting and interviewing participants were kept.

**Results:**

Challenges included taking informed consent (completing reply slips and having a 'reasonable' understanding of their participation); finding opportunities to conduct interviews; involvement of care home staff and residents' families and trying to maintain privacy during the interviews. Most residents were positive about their participation in the study, however, five had concerns either before or during their interviews. Although 15 residents seemed to feel free to air their views, three seemed reluctant to express their opinions on their care in the home.

**Conclusion:**

Although we experienced many challenges to conducting this study, they were not insurmountable, and once overcome, allowed this often unheard vulnerable group to express their views, with potential long-term benefits for future delivery of care.

## Background

In many countries older people are increasingly cared for in nursing homes or other long term care facilities towards the end of life. Three systematic reviews of research conducted in these settings have highlighted the need for empirical research in this area [1-3]. In particular, the need to represent the views of residents and their families has been highlighted[2]. There are, however, a number of challenges and methodological issues involved in conducting research in care homes for older people, which can restrict the conduct of research and prevent the views of residents from being heard.

Older people often experience a range of symptoms, including pain, fatigue and hearing or visual problems, which can severely impact all aspects of the research process, including participant recruitment, data collection, quality and analysis[4]. The increasing likelihood of cognitive impairment and dementia amongst older people is a particular challenge when it comes to taking informed consent. Whilst the importance of informed consent is widely acknowledged, the circumstances under which it is obtained amongst older people remains contentious[5]. Although it has been shown that older people may readily agree to participate in research studies, either to increase their human contact, or for the benefit of diversion, the practical aspects of conducting research in this environment can present a unique set of challenges[6]. There have also been concerns over institutionalised participants feeling an overwhelming reluctance to criticise health care professionals or feeling coerced to participate in research as a 'captive audience'[7]. In general however, it has been shown that older people at the end of life regard their participation in research as a valuable contribution to the future lives of others and that such participation can have substantial therapeutic benefits[8,9].

Poor staff compliance with research protocols, inflexibility of established routines, policies and practices in the nursing home environment, together with the potential 'gate-keeping' role of family members could create substantial obstacles to the research process. To fully represent the views of older people in care home settings, research design must be sufficiently robust to meet the strict ethical standards for vulnerable groups which govern some of the following specialist challenges; equity of participant selection, informed consent, confidentiality, risk/benefit ratio and the special protection of resident's rights[6,7].

One area, which is likely to be of great concern to residents of care homes, is preserving dignity[10]. The aim of this paper is to highlight some of the methodological challenges we experienced whilst conducting a study of the perceptions of dignity of older people living in nursing homes with a view to describing the lessons learned, responses and strategies developed and recommendations for future research study design and delivery.

## Methods

We used qualitative research methods to obtain the views on dignity of people aged 75 years and over, living in two private care homes in South East London [11]. Following local ethical and research and development approval of our protocol (King's College Hospital Research Ethics Committee: ref 07/Q0703/22), two private nursing homes in South East London were approached to participate in the study. These cared for between 40–44 residents each. The inclusion criteria for residents was aged 75 years and over. The exclusion criteria were, being unable to speak English or to provide informed consent, or too ill or distressed to take part in the study. Since we did not want to exclude the views of the oldest residents, we placed no upper age limit in eligibility.

Care home managers identified residents who they felt were eligible for the study. Since we planned to interview 20 residents, we randomly selected 15 eligible residents from each nursing home. The managers gave the selected residents our information sheets 'expression of interest' forms (all printed in large font). As some residents were later found to be unable to provide informed consent and a substantial number declined to take part, it was necessary to repeat this process several times to try to achieve the desired sample size.

Managers gave the completed expression of interest slips to the researcher (SL), who visited each home a week later to obtain written informed consent from those residents interested in taking part. Potential participants were required to have a 'reasonable' understanding of their participation in the study before informed consent was taken. They needed to (i) recall receiving the patient information sheet, (ii) give a brief account of the study and (iii) describe their involvement in the study.

Of the 86 residents in the two homes 23 (27%) were excluded by the managers, 39 (45%) did not return expression of interest forms, and six (7%) were unable to understand their participation in the study. Participants' ages ranged from 78 to 98. All but one was female, all but one was white-British, and all had multiple co-morbidities. Barthel scores[12] (ability to perform activities of daily living) ranged from total dependence (0) to nearly maximum independence (90).

Eighteen of the planned 20 interviews were eventually conducted. These were conducted by SL, who already had considerable interviewing experience, including interviews with older people and patients with serious illnesses. Interviews were in-depth and semi-structured, exploring a variety of issues including factors which either supported or undermined the participants' sense of dignity. Interview topics were closely based on recent research conducted with Canadian cancer patients[13] and lasted 45–75 minutes. At the end of each interview participants were asked how they felt about participating in the study. Two interviews were conducted with a family member present and two residents were interviewed together. To record the challenges to conducting this research SL kept detailed field notes on her experiences of recruiting and interviewing participants. These were discussed regularly at team meetings.

## Results

### The Challenges

Challenges included taking informed consent (completing reply slips and having a 'reasonable' understanding of their participation); finding opportunities to conduct interviews; involvement of care home staff and residents' families and trying to maintain privacy during the interviews.

#### Taking informed consent

Although we had intended that residents complete their 'expression of interest' forms, five residents approached by the researcher said they knew nothing about the study and that they had not completed these forms. In these instances, the researcher left residents information about the study and promised to return the following week. Of these, only one consented to take part and was eventually interviewed. This, and the lower than expected response rate, meant that we needed to invite more residents into the study, which took another two weeks.

When the researcher returned to obtain written consent, 10 of the residents who were eventually interviewed could not initially remember completing the expression of interest form or reading the information about the study, which meant she had to spend some time explaining the study. All 10 residents then had a reasonable understanding of the study, in that they were able to recall the main objectives of the study and the extent of their involvement.

Six potential participants were unable to provide informed consent to participate in the study, despite having produced completed reply slips. In these cases it was clear to the researcher that they were unable to understand their involvement in the study. So that these residents did not feel 'rejected', instead of conducting an interview, she engaged in a short neutral conversation with them and thanked them for their time. None appeared to be concerned about this or asked why they had not been included.

#### Seizing opportunities

Finding time to conduct the interviews was sometimes difficult. It was necessary to avoid busy times of the day such as mealtimes or regular visits by GPs, hairdressers, chiropodists, etc and to seize opportunities to approach residents, preferably allowing them time to recover from previous activities before commencing the interview. For example, we found it best not to interview residents after lunch as they were often tired and lethargic at this time. The researcher spent a great deal of time waiting for residents to finish activities and interviews were often postponed at a moment's notice if the resident did not feel well, had an unexpected visitor, or simply did not "feel" like participating at the moment.

#### Staff involvement

We were grateful for the help of care home staff, who were often eager to help, however, on three occasions this involved waking a resident and immediately sitting them upright. This resulted in residents feeling tired, disorientated and less likely to want to discuss the study or to be interviewed. The way in which care home staff introduced the researcher could also be problematic, as they would sometimes emphasise the name of the institution responsible for the research, resulting in some residents being confused as to who wanted to speak to them and why. Some initially thought that she was a hospital doctor visiting to discuss their health. These misunderstandings occasionally worried residents before they could be reminded of the purpose of the visit. However, once these initial problems were resolved the interviews generally progressed smoothly with the majority of residents saying that they had enjoyed taking part.

#### Privacy

Since interviews covered issues regarding the resident's care in the home, privacy was important. However in the majority of cases, the resident's door was left open or staff would enter the resident's room during the course of an interview. On two occasions staff had moved residents to a hallway and dining room where there was little or no privacy. Since most residents had mobility problems, moving them to places where they would have more privacy was time consuming and usually involved enlisting the help of busy nursing home staff.

On two occasions a member of the resident's family asked to be present for the interview. This may have had an impact on both the resident's privacy and the quality of responses given. In both cases, family members were anxious about the 'burden' the interview would place on the resident and the types of questions that would be asked. Both residents seemed to gain immense support and comfort from their relatives being present. For the relatives, it seemed also that the interview presented a chance for them to air problems that they felt would not otherwise have been raised:

"That was my main problem. I mean we used to come in, every time we'd say "Oh has she been put on the toilet?" and they'd say "Oh yeah" you know, but you know that they hadn't, and the other people (...) (whispers). But I mean you're happy though mum, aren't you?" (Daughter of Betty, a 94 year old woman who had heart failure and mild cognitive impairment)

They also often 'prompted' the participant to respond, or reminded them of events which they had forgotten. These residents then seemed to delegate all responsibility for answering certain questions to their relative, which could affect the objectivity and validity of some responses.

### Residents' feelings about taking part in the research

Of the 18 residents interviewed, 13 commented positively about their experience. Comments included enjoying having some company, being able to express their opinions freely, and feeling that they had contributed something that might benefit others in the future:

"...it makes me feel that at least somebody's interested in me." (Anne, an 84 year old woman who had a stroke)

"Well it's quite nice being talked to and expressing my opinion of what I feel and how I don't feel and you taking part in it." (Ellie, an 88 year old woman who had a stroke)

However, although they appeared to enjoy the interview, two residents said that they had initially been uncertain about how the interview would be conducted and what the experience would be like:

"Oh I've enjoyed this conversation. But I thought, the way they asked me about a week ago, that they were sending these interviewers round and I thought there'd be about three or four here, so why have they picked me out?" (Ellie)

"Well I wondered what they were going to do to me. (smiles) I hope I answered the right questions." (Jack, an 85 year old man who died the day after the interview)

Three residents were a little concerned about their 'performance' during the interview, commenting that they hoped they had answered correctly and not "talked too much". In all cases, the researcher reassured the resident several times during the course of the interview that there were no right or wrong answers to the questions, and that all views were useful and valid. Some participants took pleasure in answering the questions as they felt that this showed that they were not suffering from cognitive problems:

"I'm glad I've got the brain to answer you really." (Ellie)

"Perhaps it's because I can talk better and converse with, better than some of the people here because some of them have Alzheimer's disease and Parkinson's disease, and perhaps it's because I'm more...brainy (laughs)." (Anne)

#### Feeling free to criticise

The majority of residents (15/18) seemed to feel free to comment on their day-to-day routines and the care they received in the home. Although residents were satisfied with much of the care they received in the home, most described situations which they felt could have been handled differently by staff and suggested ways in which their care could be improved. However, three residents felt uncomfortable about voicing such criticisms:

"I found it difficult when I first came in to co-operate with the night staff. They didn't have a lot of patience. The day girls, they've been wonderful. Perhaps I shouldn't say this, should I?" (Sara, an 81 year old woman with chronic obstructive lung disease)

This could indicate concerns about reprisal from care home staff and the desire to maintain the status quo of their 'home' environment.

## Discussion

One of the most notable observations in conducting this research was the desire of residents to discuss a wide range of issues relating to dignity, which could be seen as particularly sensitive and/or emotionally challenging for older people living in a nursing home environment. These issues included the multiple losses many of them had already experienced (their homes, family and friends and their independence), as well as considering their future decline in health and death[11]. Nevertheless, most appreciated the opportunity to be heard and to make a useful contribution and were positive about taking part in the study. Their views have added to our understanding of the concerns of older people in care homes on maintaining dignity, and have led to the trial of an intervention which could help residents maintain a sense of dignity[14].

The main challenges we needed to overcome to achieve this involved obtaining informed consent, finding suitable opportunities to meet with residents, staff involvement and ensuring privacy during interviews. Although, to some extent, some of these were anticipated, we soon realised that we had underestimated the time it would take to conduct this study, and, since we had a time limit to complete it, this resulted in us conducting only 18 of the planned 20 interviews.

### Taking informed consent

Obtaining informed consent from residents is both extremely important and time consuming. Difficulty in recalling details of events from previous weeks was a problem for many residents, and one requiring some flexibility with procedures. A great deal of time and patience was needed to ensure that residents recalled and understood the study, and their role in it, before they consented to take part. Making this extra effort meant that it was possible to hear the voices of people who may otherwise have 'failed' the eligibility criteria. Conversely, we also found we had to exclude some residents who we felt did not have the capacity to provide informed consent. In some ways, the fact that managers excluded relatively few residents was reassuring as it suggests that 'gate keeping' on their part was not too much of a problem. We found ourselves, however, faced with residents who had apparently expressed an interest in taking part in the study, but we felt could not be interviewed. Taking the time to chat with them about fairly neutral topics such as the weather seemed to solve this problem. We felt that they had forgotten about the study and enjoyed the company of an extra visitor.

For us, the lesson learned was that it is important to take as much time as necessary to check the understanding of participation in a gentle, non-threatening way, and to be very tactful when excluding people who cannot provide informed consent. Researchers have to be particularly patient, and the extra time and training for this needs to be built into the design of the research. It may be especially helpful for the research team to develop set protocols for researchers on how to respond and handle a variety of potential responses, to ensure uniformity and consistency. It is also important to ensure that all information given to potential study participants is clearly written and in an appropriate format for those with visual impairment. We found that residents were often suspicious of strangers and found disruptions to their expected daily routine a little unsettling. In future studies we plan to leave residents a card with the date, time and length of the next visit with a photograph of the researcher(s). This will also have clear information about which institution they come from.

### Seizing opportunities and staff involvement

Although we were well aware that there would be times when residents would not be free to meet with the researcher, we were surprised at how few and how unpredictable opportunities would be. We needed to be sensitive to the needs of residents and respect their decision not to be interviewed at the agreed time, without asking them to justify their decision. Although staff were generally helpful, they were usually very busy, and sometimes the researcher needed to wait some time to be introduced to a resident or for them to be moved to a place of privacy. We found that a great deal of flexibility and reciprocity was needed to conduct research in this setting. The time spent developing relationships with staff and on discovering established routines and practices in the homes was time well spent.

### Ensuring privacy and reassuring residents

It was unusual for residents to be concerned about being critical of the care they received in the home, despite the fact that it was sometimes difficult to maintain privacy during the interviews. It is possible that residents who seemed uncomfortable about expressing negative views had never liked to complain, or it could be that they have had negative experiences after complaining in the past. The number of unexpected interruptions certainly didn't help them feel at ease to express such views. We found that one of the factors that eroded a residents' sense of dignity was loss of privacy[11] and concerns about loss of privacy have been raised in other studies of older people living in care homes[15]. Ensuring confidentiality and privacy is usually outlined in study protocols and scrutinised by ethics committees. However, it is important to communicate the importance of maintaining privacy to care home staff and to continually remind residents that their interviews are confidential and would not be shared with care home staff. However, should they prefer to have someone else present during the interview, the interviewer needs to be skilled in ensuring that the resident still has the opportunity to be heard. Older people can become used to not being heard and loose confidence in voicing their opinions. Most have never taken part in research or been interviewed before. It is perhaps not surprising that some find this a little daunting. We found that regularly emphasising the 'informality' of the interviews process and a friendly and patient interviewer who gave them encouragement and reassurance throughout the interview helped them to adjust to and enjoy this new experience.

The main limitations of this study relate to recruitment (outlined previously) and the fact that only one researcher conducted the interviews. The interviewer cannot completely avoid bias, and the interpretation of participants' experience and consideration of the data by only one researcher is limited. Had more than one interviewer been used, and inter-rater reliability measures included, the interpretation would have been more robust.

## Conclusion

Although the challenges experienced throughout this study were numerous, they were not insurmountable. The key lessons we learned from our experiences were to have patience and allow plenty of time. The extra time and costs are a small price to pay to hear the views of this under-represented section of society.

## Competing interests

The authors declare that they have no competing interests.

## Authors' contributions

SH and IJH designed the study. SL conducted the interviews with residents. SH and SL co-wrote the manuscript, and IJH critically revised it. All authors read and approved the final manuscript.

## Pre-publication history

The pre-publication history for this paper can be accessed here:


